# Disorders associated with malabsorption of iron: A critical review

**DOI:** 10.12669/pjms.316.8125

**Published:** 2015

**Authors:** Muhammad Saboor, Amtuz Zehra, Khansa Qamar, Moinuddin

**Affiliations:** 1 Dr. Muhammad Saboor, Ph.D, MLS(ASCP^i^)SH. Baqai Institute of Hematology, Baqai Medical University, Karachi, Pakistan; 2 Dr. Amtuz Zehra, MBBS. Department of Pathology, Baqai Medical University, Karachi, Pakistan; 3 Dr. Moinuddin FRCP (C), FRCP (E). Baqai Institute of Hematology, Baqai Medical University, Karachi, Pakistan; 4 Khansa Qamar, M.Phil. (Hematology). Baqai Institute of Hematology, Baqai Medical University, Karachi, Pakistan

**Keywords:** Iron deficiency anemia, Malabsorption, Gastrointestinal tract

## Abstract

Malabsorption is a disorder of the gastrointestinal tract that leads to defective digestion, absorption and transport of important nutrients across the intestinal wall. Small intestine is the major site where most of the nutrients are absorbed. There are three main mechanisms of malabsorption; premucosal, mucosal and postmucosal. Premucosal malabsorption is the inadequate digestion due to improper mixing of gastrointestinal enzymes and bile with chyme. This could be because of surgical resection of the small intestine or a congenital deficiency of the enzymes and bile responsible for digestion e.g. postgastrectomy, chronic pancreatitis, pancreatic cancer, cystic fibrosis, gallstones, cholangitis etc. Mucosal malabsorption occurs in celiac disease, tropical sprue, Crohn’s disease etc. Postmucosal condition arises due to impaired nutrients transport e.g. intestinal lymphangiectasia, macroglobulinemia etc. Disorders of malabsorption lead to decreased iron absorption and produce iron deficiency anemia.

Using the index terms malabsorption, postgastrectomy, chronic pancreatitis, pancreatic cancer, cystic fibrosis, gallstones, cholangitis, celiac disease, tropical sprue, Crohn’s disease intestinal lymphangiectasia, macroglobulinemia and iron deficiency anemia the MEDLINE and EMBASE databases were searched. Additional data sources included bibliographies and references of identified articles.

## INTRODUCTION

Malabsorption is the alteration in the functions of gastrointestinal tract (GIT) that subsequently affects digestion, absorption and transport of body’s essential nutrients across the intestinal wall. Small intestine is the major site where absorption of various types of ingested nutrients (fats, carbohydrates and proteins), micronutrients (iron, vitamins, minerals), water and electrolytes occurs.^1^,^2^ Small intestine has a specialized mucosal surface (villous and micro villous) for the uptake of nutrients. This mucosal surface secretes numerous digestive enzymes. It also contains a network of blood vessels and lymphatics that gives easy access for the nutrients to reach the bloodstream. A number of factors are involved in proper digestion and absorption. Some of these are mechanical mixing, digestive enzyme production and their activity, peristaltic movements of GIT, blood supply and normal microbial environment. Alteration in any of these components can lead to malabsorption of ingested nutrients.^3^-^5^

### Epidemiology of malabsorption

IDA rarely occurs in isolation. It is usually present in conjunction with other conditions such as hookworm infestation, nutritional deficiencies, malabsorption, hemoglobinopathies etc.^6^,^7^ Incidence of iron deficiency anemia (IDA) secondary to GIT diseases in adult men and postmenopausal women is approximately 2-5% in developed countries.^8^ In Pakistan, there is scanty data regarding iron deficiency anemia associated with GIT disorders. Studies have shown that the incidence of nutritional anemia associated with GIT diseases in women of reproductive age is about 5% and in postmenopausal women it is 6.8% in Pakistan.^9^,^10^

### Causes of malabsorption

Etiologically, malabsorption may be caused by three mechanisms. These are premucosal (luminal), mucosal and postmucosal (postabsorptive). Premucosal causes lead to maldigestion while mucosal and postmucosal conditions are associated with actual malabsorption.^11^ Causes of malabsorption are listed below;

### Premucosal

- ***Inadequate digestion:*** Postgastrectomy, chronic pancreatitis, Cystic fibrosis, Pancreatic resection, Zollinger-Ellison syndrome
- ***Deficient bile salt:*** Obstructive jaundice, gall stone, terminal ileal resection

### Mucosal

- ***Primary mucosal abnormalities:*** Celiac disease, tropical sprue, Whipple’s disease, amyloidosis, Giardiasis, *H.pylori* infection
- ***Inadequate absorption in small intestine:*** Crohn’s disease, intestinal resection, jejunoileal bypass

### Postmucosal

- ***Lymphatic obstruction:*** Intestinal lymphangiectasia, malignant lymphomas, macroglobulinemia

Causes of malabsorption show characteristic geographical distribution. Celiac disease, Crohn’s disease, cystic fibrosis and intestinal lymphangiectasia are commonly encountered in the West. Tropical sprue, parasitic infestation, intestinal tuberculosis and primary immunodeficiency syndrome are the frequent causes of malabsorption in the East.^12^

Premucosal malabsorption is the result of inadequate digestion. It occurs due to insufficient mixing of chyme with bile salts and enzymes of gastrointestinal tract (pancreatic and intestinal disorders) e.g. chronic pancreatitis, pancreatic cancer, cystic fibrosis, gallstones, cholangitis and biliary atresia are associated with premucosal malabsorption.^1^,^5^ Mucosal conditions result in a reduced absorptive area. These include celiac disease, inflammatory bowel syndrome and Whipple’s disease. Post mucosal causes arise due to those factors that hinder nutrient transport. Vascular or lymphatic obstructions are examples of post mucosal causes of malabsorption.^5^,^12^

### 1. Premucosal causes: Diseases related topre mucosal malabsorption are described below

### Chronic pancreatitis

Chronic pancreatitis is an irreversible progressive disease characterized by destruction of the pancreatic secretory parenchyma that leads to fibrosis of the pancreatic tissue.^13^ Main etiological factors that cause chronic pancreatitis are alcohol consumption, smoking, pancreatic duct obstruction, hereditary pancreatitis and recurrent acute pancreatitis. It’s pathogenesis is variable.^14^ Necrosis and fibrosis causes decreased release of enzymes causing malabsorption. It is usually diagnosed by ultrasound, computed tomography (CT) scan or magnetic resonance cholangiopancreatography (MRCP).

### Cystic fibrosis

Cystic fibrosis is an autosomal recessive disorder that affects lungs, liver, pancreas and gastrointestinal tract. In this disease there is mutation of the gene which forms the protein cystic fibrosis transmembrane conductance regulator (CFTR).^15^ CFTR is a channel protein present on the cell membrane, its main function is to regulate the movement of chloride and bicarbonate ions and water across the cell. In cystic fibrosis this protein is altered. In GIT this altered protein causes decreased secretion of chloride ion and hyperabsorption of water resulting in thick mucous fecal material. This causes intestinal obstruction which results in malabsorption.^16^

### Zollinger-Ellison syndrome

Zollinger-Ellison syndrome is a disorder characterized by excessive secretion of gastrin hormone. Mostly, it is a secondary disease caused by a tumor in pancreas (gastrinoma), duodenum or abdominal lymph nodes which causes increased secretion of gastrin. Gastrin is a hormone released from G cells present in pancreas, stomach and duodenum.^17^ This hormone acts on parietal cells of stomach and releases hydrochloric acid (HCl). In the presence of gastrinoma, gastrin production is increased by the tumor which causes increased secretion of HCl. This causes gastric ulceration and gastro–esophageal reflux resulting in malabsorption.^18^ It is diagnosed by endoscopy and secret in test.

### Obstructive jaundice

Jaundice, also known as icterus, is a yellowish pigmentation of the skin and mucous membrane caused by high blood bilirubin level. Obstructive jaundice, as the name suggests, is due to the obstruction in the biliary tract.^19^ Main cause of obstructive jaundice is gallstone. Other causes include carcinoma of the head of the pancreas, carcinoma of the bile duct or biliary stricture. Bile is conjugated in liver and is either stored in the gall bladder or it enters the second part of duodenum through biliary tract for digestion. Obstruction in the biliary tract inhibits the entry of bile in the intestine and its participation in the digestion causing malabsorption. Other sign and symptoms are dark coloured urine as conjugated bilirubin is excreted through the urine and pale stools due to the absence of urobillinogen which is formed when billirubin is reduced by intestinal flora.^20^

### 2. Mucosal causes

### Celiac disease

Celiac disease, also known as gluten sensitive entropathy, is a hereditary immune mediated disease. It results from inappropriate cell mediated immune response to gluten-containing diet (wheat, rye and barley) in genetically susceptible individuals. Activation of cell mediated immune response causes intestinal tissue damage.^21^ In celiac disease, prevalence of IDA is approximately 46%. Its incidence is higher in adults than in children. Diagnosis of celiac disease is based on clinicopathological studies that include serological testing, small bowel mucosal biopsies and unambiguous response to gluten free diet.^22^

### Inflammatory bowel disease (IBD)

Iron deficiency anemia in IBD occurs due to chronic blood loss from GIT and poor absorption through the small intestinal mucosa. IBD includes Crohn’s disease and ulcerative colitis. Prevalence of iron deficiency anemia in IBD is about 63%. Diagnosis of IBD is based on a combination of clinical, endoscopic and histological features.^23^

### H. pylori infection

*H. pylori* infection like other malabsortion diseases may also cause IDA. *H. pylori* usually cause gastric ulcer or gastric malignancy. Studies have shown that it may cause extra gastric disease such as *H. pylori* associated anemia. Mechanism of *H. pylori* associated anemia is unknown. *H. pylori* associated iron deficiency anemia is mainly present in those individuals who have increased demand for iron as in children and in pregnant, postpartum and premenopausal women.^24^

### Tropical sprue

Tropical sprue is one of the commonest causes of malabsorption especially in adults. It is a disease of tropical and subtropical regions i.e. South Asia (China, India, Pakistan). A few decades ago, tropical sprue was considered as an endemic disease in soldiers and prisoners in these regions. Lately, the incidence of tropical sprue has markedly decreased. There are certain reasons for the reduced incidence and eradication of this disease. Some of these are improvement in socioeconomic status, better sanitation, awareness of water borne diseases, proper diagnosis of bacterial and viral infections, increased use of antibiotics and probiotics.^25^,^26^

### Whipple’s disease

It is a rare multi systemic infectious disease. It is caused by a bacterium, Tropheryma whipplei. It was first described by George Whipple in 1907. It is primarily dominated by malabsorption but it also affects other parts of the body including lungs, brain, heart, eyes and skin.^27^ It is postulated that the bacteria enter the body through oral ingestion and reside in intestinal villi. They subsequently enter the macrophages present in the lamina propria of the villi and replicate there. After apoptosis the bacteria are released and spread through lymphatic route throughout the body. Its major signs and symptoms are diarrhea, weight loss, joints pain and arthritis. It is diagnosed by duodenal biopsy.^28^

### Amyloidosis

It is a rare disease which occurs due to accumulation of misfolded proteins known as amyloid in the organs such as heart, kidney and gastrointestinal tract. The proteins when formed sometimes fold inappropriately and become insoluble. They cannot be broken down by proteolysis with resultant accumulation and deposition in various organs.^29^ These proteins alter the function of the organs. In the gastrointestinal tractamyloid material attaches to the intestinal villi and causes malabsorption. Sign and symptoms depend on the organ affected. Diagnosis is made on biopsy.^30^

### Giardiasis

Giardiasis is a disease of intestine caused by a parasite, Giardia lamblia. It is transmitted through oral-fecal route.^31^ After ingestion, the giardia cysts undergo excystation and attach to the enterocytes and stimulate the process of apoptosis. It also breaks the enterocyte barrier leading to malabsorption of sodium and glucose and hypersecretion of chloride causing diarrhea.^32^ Other signs and symptoms are malaise, steatorhhea (fat in feces) and generalized weakness. Diagnosis is mostly made on stool examination or blood serology.

### Surgical resections

Surgical procedures such as postgastrectomy, pancreatic resection, terminal ileal resection, jujenoileal bypass and intestinal resections cause premucosal or mucosal malabsorption due to removal of a part of intestine.

### 3. Postmucosal causes

### Intestinal lymphangiectasia

Lymphangiectasia is the dilatation of the lymph vessels. In intestinal lymphangiectasia there is dilatation of the lymph vessels present in the small intestine. Function of these vessels is to carry fats and proteins absorbed by the small intestine to the bloodstream. Dilatation of these vessels causes backflow and leak fats and proteins back again into the intestinal lumen resulting in malabsorption. These leaky channels cause hypoalbuminemia and leucopenia.^33^ It is diagnosed by biopsy.

### Malignant lymphoma

Malignant lymphoma is a tumorous condition of the lymphatic system. In this condition lymph nodes are enlarged and solid tumorous are found in the lymphatic system causing obstruction in the lymph vessels and also affecting the lymphatic vessels of the intestine.^34^ This causes decreased absorption and increased leaking of digested food in the lumen leading to malabsorption.

### Macroglobulinemia

Macroglobulinemia is a condition with increased concentration of macroglobulins in the blood. It is associated with plasma cell dyscrasias. Macroglobulinemia causes hyperviscosity syndrome and lymphoplasmatic infiltration of the tissues and bone marrow.^35^ Association of macroglobulinemia and malabsorption is rare. It occurs due to the lymphoplasmatic infiltration in the small intestine. Biopsy is done to diagnose this disease.^36^

## CONCLUSION

Malabsorption of iron is an uncommon cause of iron deficiency anemia. Most of the cases are secondary to some underline pathological process in the gastrointestinal tract. However cases of primary malabsorption of iron do exist although their incidence is rather small.
